# Effect of Bedtime Melatonin Administration in Patients with Type 2 Diabetes: A Triple-Blind, Placebo-Controlled, Randomized Trial

**DOI:** 10.22037/ijpr.2019.112011.13485

**Published:** 2019

**Authors:** Amir Farrokhian, Maryam Tohidi, Noushin Sadat Ahanchi, Davood Khalili, Mahtab Niroomand, Arash Mahboubi, Arash Derakhshi, Mohammad Abbasinazari, Farzad Hadaegh

**Affiliations:** a *Department of Clinical Pharmacy, School of Pharmacy, Shahid Beheshti University of Medical Sciences, Tehran, Iran* *. *; b *Prevention of Metabolic Disorders Research Center, Research Institute for Endocrine Sciences, Shahid Beheshti University of Medical Sciences, Tehran, Iran* *. *; c *Department of Biostatistics and Epidemiology, Research Institute for Endocrine Sciences, Shahid Beheshti University of Medical Sciences, Tehran, Iran* *. *; d *Internal Medicine Department, Endocrinology Division, Shahid Beheshti University of Medical Sciences, Tehran, Iran*; e *Food Safety Research Center, Department of Pharmaceutics, School of Pharmacy, Shahid Beheshti University of Medical Sciences, Tehran, Iran* *.*

**Keywords:** Melatonin, Diabetes mellitus, Glycemic status, Dietary supplement, Melatonin level

## Abstract

Melatonin is widely available as over the counter product. Despite promising effects of melatonin supplementation on glycemic control, there is a significant heterogeneity between studies. The current study aimed at determining the effect of melatonin on fasting blood glucose (FBG), insulin resistance/sensitivity indices, glycosylated hemoglobin A1c (HbA1c), and high sensitivity C-reactive protein (hs-CRP) among type 2 diabetes mellitus (T2D) population during 8 weeks in a randomized, triple-blind, placebo-controlled trial. Thirty four subjects with the mean age ± standard deviation of 57.74 ± 8.57 years and 36 subjects with the mean age of 57.61 ± 9.11 years were allocated to 6 mg nightly melatonin and placebo groups, respectively. Melatonin and placebo groups were matched by age, gender, body mass index, and duration of diabetes. Also, there was no significant difference in laboratory findings except for HbA1c, which was lower in the placebo group (7.00 ± 0.89% vs 7.60 ± 1.47%, *P*=0.042). After trial completion, the increase of serum levels of melatonin was greater in the intervention than the placebo group (3.38 ± 1.33 vs 0.94 ± 1.28 ng/L, *P*=0.192). Moreover, compared to placebo group, among melatonin users, homeostasis model assessment of insulin resistance (HOMA1-IR) tended to be unfavorable at the end of follow-up [-0.51 (-1.76-0.81) vs. 0.28 (-1.24-1.74), *P*=0.20]; the similar trend was also shown for insulin sensitivity index (HOMA1-S) [2.33 (-3.59-12.46) vs. -2.33 (-10.61-9.16), *P*=0.148]. No differences were observed in FBG, HbA1C, and hs-CRP changes between the trial groups. The current study did not support the improving effect of melatonin on glucose homeostasis.

## Introduction

Type 2 diabetes mellitus (T2D) is a highly prevalent endocrine disorder, particularly among West Asian countries around the Persian Gulf (1). Both types of diabetes can lead to microvascular complications, including nephropathy, retinopathy and neuropathy, and macrovascular disorders such as stroke, ischemic heart disease and peripheral vascular disease. The premature morbidity, mortality, reduced life expectancy besides costs of diabetes make it an important public health condition (2). Some new studies on diabetes care are focused on treatments that can improve the health status of people with T2D. Recently, new treatment that either affect physiologic axis such as cabergoline (3) or new agents such as vitamin D3 (4) are examined.

Melatonin produced by different tissue, mainly pineal gland, undergoes circadian fluctuation that peaks at night (80-100 pg/mL) and decreases during the day (10-20 pcg/mL) in mammalians (5). Receptors of melatonin, 1A and 1B, are both expressed in pancreatic islets (6). Melatonin is a pleiotropic hormone responsible for different functions including time regulation of circadian rhythms (7), stabilization of sleep/awake cycle (8), reduction of oxidative stress, increase in expression of antioxidant enzymes (9), impact on vascular constriction and dilation (10), modulation of immune system, and decrease of inflammation (11).

Nowadays, melatonin is widely available over the counter in many countries; thus, it is necessary to highlight the importance of more knowledge about its effects on metabolic parameters (12). Several animal studies indicate that melatonin has beneficial effects on glycemic control in diabetes via improvement in glucose hemostasis and diabetes pathogenesis (13, 14), amelioration of the negative effects of both nitrogen and oxygen oxidative stresses and increase in expression and function of antioxidant enzymes (9, 15-18), and regulation of insulin secretion from isolated islets (6). Among few trials that assessed the effects of melatonin on glycemic control, limited studies are conducted on T2D human population. Saad *et al.,* reported positive effect of melatonin plus zinc on fasting and postprandial plasma glucose (19). In another study, Rezvanfar *et al.,* reported better control of T2D with 6 mg bedtime administration of melatonin, based on glycosylated hemoglobin A1c (HgbA1c) and fasting plasma glucose (FPG). The present study aimed at evaluating the effect of melatonin on glycemic control and insulin sensitivity/resistance indices in patients with T2D in a triple-blind, randomized, controlled trial (RCT).

## Method

*Design overview and patients*

This triple-blind, placebo-controlled, parallel-group randomized clinical trial was conducted from October 2017 until December 2018. This study was approved by the ethics committee of Research Institute for Endocrine Sciences, Shahid Beheshti University of Medical Sciences (code: IR.SBMU.ENDOCRINE.REC.1396.424) and was registered in the Iranian Registry of Clinical Trials (code: IRCT20121021011192N4).

Participants were enrolled from a diabetes clinic in a teaching hospital and a private office. Inclusion criteria were: patients above 18 years old with diagnosis of T2D according to American Diabetes Association (ADA) guideline, 2018 (20) with normal hepatic and renal function. Written consent was signed by all participants.

Subjects with the following conditions were excluded from the study: Pregnancy, breast feeding, shift workers, history of hypersensitivity to melatonin, medication abuse and drug addiction or current addiction, coagulopathy, history of epilepsy or current active disease, history of uncontrolled depression or current active disease, current users of nifedipine, fluvoxamine, and melatonin. If any serious adverse events occurred during the intervention, patient was excluded from the study.

*Intervention*

Patients were randomly divided with 1:1 ratio into the control and intervention groups by Microsoft Excel software. Block randomization was implemented. After the collection of the 1st blood samples, bedtime administration of 6 mg melatonin (NatureMade®, USA) or placebo with identical appearance was initiated. The placebo contained cellulose instead of melatonin and both the tablets had the same size, shape, color, odor and package. The 2nd blood sample was taken after about 60 consecutive days from all patients. During the study, adherence to medication was evaluated by at least two telephone calls, and complications or any adverse events were recorded. 

Participants, principle investigator, healthcare providers (physicians), medication provider, data collectors, and outcome assessors were all blind to the study grouping. A responsible co-researcher who was not involved in running the study organized melatonin and placebo tablets.

*Blood sampling and measurements*

Baseline and follow-up blood samples were obtained after 12 hours overnight fasting at 8:00 to 9:00 AM. Samples were collected in K_2_EDTA (dipotassium ethylenediaminetetraacetic acid) for HbA1c and anticoagulant-free tubes for fasting blood glucose (FBG), melatonin, high sensitivity C-reactive protein (hs-CRP), and insulin measurements. 

HbA1c levels were measured on the sampling days by an enzymatic method using Hitachi 911 Chemistry Analyzer (Roche Diagnostics, GmbH, Mannheim, Germany), with intra- and inter-assay coefficients of variation (CVs) of 1.0% and 1.1%, respectively. Serum samples were stored at -80°C for other measurements. Serum glucose levels were measured by enzymatic colorimetric method (Pars Azmun Co. Tehran, Iran) on Pictus 700 Clinical Chemistry Analyzer, Diatron MI Plc (Budapest, Hungary); intra- and inter-assay CVs were 1.1% and 1.7%, respectively. Serum melatonin and hs-CRP were measured by enzyme immunometric assays using commercial kits (ZellBio® GmbH, Ulm, Germany) and the Sunrise ELISA reader (Tecan Co. Salzburg, Austria) with intra- and inter-assay CVs of 3.2% and 3.8%, respectively, for melatonin, and 2.1% and 4.9%, respectively for hs-CRP. Fasting serum insulin was measured by the electrochemiluminescence immunoassay (ECLIA), using Roche Diagnostics Kit and the Roche/Hitachi Cobas e-411 Analyzer (GmbH, Mannheim, Germany) with intra- and inter-assay CVs of 1.0% and 1.8%, respectively. Original homeostasis model assessment (HOMA1) were calculated for insulin resistance (HOMA-IR) as HOMA1-IR = fasting insulin (IU/mL) × fasting glucose (mM/L)]/22.5, for beta cell function (HOMA-B) as HOMA1-B = [20 × fasting insulin (IU/mL)]/[fasting glucose (mM/L)-3.5], and for insulin sensitivity (HOMA-S) as HOMA1- S = 1/HOMA1-IR × 100; updated HOMA models (HOMA2) were calculated using fasting glucose and fasting insulin in a steady-state condition by HOMA calculator for specific insulin available on http://www.dtu.ox.ac.uk/homacalculator (21).

*Data and statistical analyses*

Continuous variables are expressed as the mean ± standard deviation (SD) or interquartile range for normal and skewed distributed variables, respectively, and percentages for categorical variables. Comparisons of baseline characteristics between melatonin and placebo groups were performed using independent samples t test, Mann–Whitney test, and Pearson’s Chi-squared test as appropriate. Means of variables were compared within groups using the paired t test and Medians of variables were compared within groups using the Wilcoxon test for normal and skewed distributed variables. The changes of marginal means of the outcome variables were reported applying ANCOVA, adjusted for the baseline level of each variable and Mann–Whitney test for variables with a non-normal distribution. Statistical analyses were performed by SPSS Software (V.20) and *P*≤0.05 was considered to be to be statistically significant.

## Results

**Figure1:**
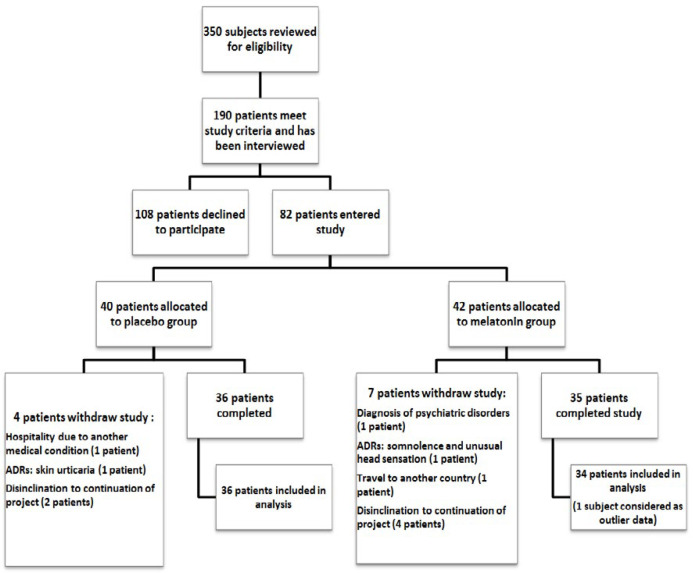
Summary of patient flow diagram, ADRs: Adverse Drug Reactions

About 350 subjects were screened for eligibility criteria. Just 190 patients met the study criteria and were interviewed in order to obtain their constant for participation in the study. Finally, 82 patients were enrolled in the study and allocated to melatonin or placebo group. However, 36 and 35 patients completed the study in placebo and melatonin groups, respectively. One patient in the placebo group was not considered in the statistical analysis as outlier data (flow of the study-Figure.1).

Baseline characteristics of the study participants are shown in Table 1. Melatonin and placebo groups were matched by age, gender, body mass index, and duration of diabetes. Also, there was no significant differences in laboratory findings except for HbA1c between the melatonin and placebo subjects (7.00 ± 0.89% vs 7.60 ± 1.47%, respectively, *P*=0.042), and in the current medications except for metformin use (100% vs 86.11%, respectively; *P*=0.024). 

As shown in Table 2, comparing before and after melatonin trial, serum melatonin levels increased significantly in the intervention arm (*P*=0.036), however, no change was noticed in the placebo group. FBG levels in the placebo arm decreased significantly at the end of trial (153.19 ± 42.70 vs 137.72 ± 28.65 mg/dL, *P *= 0.014).

Changes of laboratory parameters after melatonin trial between the intervention and placebo groups are shown in Table 3. At the end of follow-up, among melatonin users compared to placebo subjects, HOMA1-IR increased [0.28 (-1.24-1.74) vs. -0.51 (-1.76-0.81), *P*=0.200] and HOMA1-S decreased [-2.33 (-0.61-9.16) vs. 2.33 (-3.59-12.46), *P*=0.148]; however, no significant difference was observed in the other parameters.

*Safety*

Somnolence was the most common adverse event reported both in melatonin and placebo groups. Just one subject withdrew the study due to the occurrence of adverse effects (somnolence and unusual head sensation) in the melatonin intervention arm. Table 4 shows details of adverse events reported by the participants.

## Discussion

During an eight-week RCT among patients with T2D, it was observed that adding 6 mg/day nightly melatonin to the background pharmacological management of patients did not have any favorable impact on HbA1c, FBG, insulin resistance/sensitivity indices, beta cell function, and hs-CRP levels. Actually, compared to the placebo group, in melatonin users, HOMA1-IR and HOMA1-S tended to be unfavorable at the end of follow-up. 

Data regarding the effect of melatonin on diabetic population are limited and controversial. Only two studies evaluated the effects of melatonin on both HbA1c and FPG among patients with T2D. One study with intervention of 10 mg melatonin plus zinc for 30 days indicated decrease both in FPG and HbA1c levels (19); however, it is not possible to attribute these results to melatonin per se. Similarly, Rezvanfar *et al.,* mentioned that melatonin supplementation (6 mg/day) for 12 weeks in a before-after study resulted in about 10.7% mg/dL and 0.34% decrease in FBG and HbA1c, respectively (22). Other RCT conducted on patients with T2D and chronic heart disease (CHD) found that during a 12-week-trial, adding 10 mg/day melatonin decreased levels of FPG (-29.4 ± 49.0 mg/dL) (23). Finally, a recent meta-analysis on the impact of melatonin on glycemic parameters concluded that melatonin had beneficial effects on FPG. The pooled findings indicated that melatonin supplementation significantly reduced FBG about 6 mg/dL (MD = –6.34; 95%CI: –12.28, –0.40; *P*= 0.04; I^2 ^= 65%) without any impact on HbA1c. Importantly, the authors claimed that the results of publication bias were statistically significant for this positive effect (24). The current study, however, did not confirm the favorable effect of melatonin on FPG level.

Regarding changes in insulin resistance and sensitivity as well as insulin level per se, the current study data analysis did not find any differences between placebo and melatonin arms for HOMA parameters including HOMA1-IR, HOMA1-B, HOMA1-S, and insulin levels. Actually, in the current study RCT, the level of insulin resistance/sensitivity indices tended to be worsening among melatonin users. In line with the current study findings, acute administration of 5 mg melatonin to healthy female patients resulted in the impairment of glucose tolerance primarily by decreasing insulin release in morning and reducing insulin sensitivity in the evening (25). Moreover, among subjects with metabolic syndrome, a 10-week administration of bedtime melatonin (8 mg/day) showed modest, but not significant, decrease in waist circumference, triglyceride, and high lipoprotein cholesterol associated with slight worsening of FPG (26). In contrast to these studies, a trial conducted on patients with T2D and CHD, however, showed decreasing level of insulin associated with improving insulin resistance/sensitivity indices (23). The complex and contradictory impact of melatonin on glucose homeostasis between studies might be attributable to different genotypic background of study population. For example, inhibitory action of melatonin on insulin secretion was observed in human pancreatic islets of carriers of the common variant rs10830963 (a risky allele) more than in the islet of patients without this variant (12). Hence, the worsening of insulin resistance following acute administration of melatonin both in morning and evening (25), in contrast to its favorable impact during long-term treatment on patients with nonalcoholic steatohepatitis and the ones with insomnia might be attributable to the status of the rs 10830963 locus, the issue which was not addressed in these studies (27, 28). Other sources of heterogeneity between studies were related to different study designs (for example, double-blind, placebo-controlled trial vs. open labeled trial), different dosages of melatonin (high vs. low), and different baseline values of studied variables. 

In the current study, no favorable effect on hs-CRP, as an inflammatory marker, was observed for melatonin. However, two other studies, using higher dosages of melatonin (10 mg/day) showed a reduction in serum hs-CRP levels (23, 29). Recently, a meta-analysis of RCTs showed no favorable impact of melatonin among studies using lower dosages of melatonin (i e, 6 mg/day) or studies with a duration of less than 8 weeks (30); the issues that supported the neutral impact of melatonin on hs-CRP among the current study participants.

Importantly, melatonin pharmacokinetic parameters vary in different investigations. Inter-individual variation and its impacts on biopharmaceutical variables are also reported. The exact intestinal absorption fraction of oral melatonin in humans is not established. Wide range of bioavailability after oral administration, from 3% to 56%, is observed in different studies. It may either cause low absorption from the gastrointestinal tract, an extensive first-pass metabolism, or a combination of both (31, 32). To the best of authors` knowledge, the current study was the first and only trial that overcomes one of the main limitations of previous ones, reporting significant increase in melatonin serum levels in the intervention group at the end of trial, confirming complete adherence.

No serious adverse effect was reported by supplemented participants in the present study, a consonant finding with previous studies (33, 34). Somnolence, as most common reported side effect of this supplement, easily manages by bedtime administration. Overall, melatonin is a safe and highly tolerable agent.

The results of the current study should be interpreted in the light of several limitations. First, although the study was adequately powered to examine the primary objective (HbA1c), non-significant P-values in the secondary outcomes might be explained by relatively small sample size. Second, the current study results might not be applicable to studies that applied higher doses of melatonin during longer trials; however, using melatonin in 8 mg dosage for 10 weeks was also associated with worsening of plasma level of glucose (26). Third, diet and exercise were not controlled during the intervention, which might affect the results.

In summary, the current study findings did not support the improving effect of melatonin on glucose homeostasis among subjects with T2D; actually, some deteriorating effects on insulin resistance/insulin sensitivity were highlighted.

**Table1:** Baseline characteristics of the study participants

	**Melatonin group (n = 34)**	**Placebo group (n = 36)**	***p *** ** value**
Age (years)	57.74 ± 8.57	57.61 ± 9.11	0.830
Sex—female (%)	17 (50.00)	16 (44.44)	0.642
BMI (kg/m²)	29.33 ± 4.53	27.60 ± 5.05	0.136
Duration of disease (years)	11.10 ± 7.06	11.21 ± 7.25	0.949
Melatonin (ng/L)	35.43 ± 13.85	36.06 ± 15.07	0.856
HbA1c (%)	7.60 ± 1.47	7.00 ± 0.89	0.042
FBG (mg/dL)	151.09 ± 38.62	153.19 ± 42.70	0.830
Hgb(g/dL)	14.98 ± 1.64	14.73 ± 2.10	0.576
Insulin (μU/mL)	12.05 (6.32-17.44)	12.34 (6.56-25.07)	0.505
HOMA1-IR	4.34 (2.77-6.38)	4.09 (2.28-9.49)	0.671
HOMA1-b	49.46 (34.30-94.80)	54.06 (26.57-97.37)	0.359
HOMA1-s (%)	23.08 (15.66-36.10)	24.39 (10.56-43.68)	0.879
HOMA2-IR^c^	1.75 (0.99-2.47)	1.73 (0.96-3.63)	0.515
HOMA2-b (%)	46.55 (34.80-82.30)	46.55 (30.65-87.10)	0.484
HOMA2-s (%)	56.90 (40.35-100.90)	57.80 (27.50-103.97)	0.968
Hs-CRP (ng/mL)	2994.59 (1390.82-4117.30)	2213.11 (884.43-3984.21)	0.377
Medications (%)			
*Insulin*	15 (44.11)	14 (38.88)	0.657
*Metformin*	34 (100)	31 (86.11)	0.024
*Sulfonylurea*	14 (41.17)	12 (33.33)	0.497
*Meglitinide analog*	1 (2.94)	1 (2.78)	0.967
*Thiazolidinediones*	0 (0)	0 (0)	a
*GLP-1 rec agonists*	0 (0)	0 (0)	a
*DPP-4 inhibitors*	17 (50.00)	16 (44.44)	0.642
*ACEi/ARBs*	18 (52.94)	18 (50.00)	0.806
*CCBs*	4 (11.76)	4 (11.11)	0.932
*Diuretics *	6 (17.64)	7 (19.44)	0.847
*Beta blocker*	14 (41.17)	9 (25.00)	0.150
*Statin*	28 (82.35)	33 (91.66)	0.245
*ASA*	21 (61.76)	16 (44.44)	0.147
*Nitrate*	1 (2.94)	2 (5.55)	0.589


**Table2:** Comparison of measured markers within groups before and after melatonin intervention trial

**Variables**	**Melatonin group**		***p *** **value**	**Placebo group**		***p *** **value**
	***Before***	***After***		***Before***	***After***	
Melatonin (ng/L)	35.43 ± 13.85	38.70 ± 14.31	0.036^a^	36.06 ± 15.07	37.04 ± 14.73	0.414^a^
HbA1c (%)	7.60 ± 1.47	7.32 ± 0.95	0.278^a^	7.00 ± 0.89	7.08 ± 1.07	0.344^a^
FBG (mg/dL)	151.09 ± 38.62	138.12 ± 31.05	0.124^a^	153.19 ± 42.70	137.72 ± 28.65	0.014^a^
Insulin (μU/mL)	12.05 (6.32-17.44)	13.15 (8.06-19.14)	0.139^b^	12.34 (6.56-25.07)	9.91 (7.21-23.38)	0.706^b^
HOMA1-IR	4.34 (2.77-6.38)	4.20 (2.53-5.86)	0.651^b^	4.09 (2.28-9.49)	3.13 (2.50-7.34)	0.220^b^
HOMA1-b	49.46 (34.30-94.80)	59.77 (33.02-119.61)	0.203^b^	54.06 (26.57-97.37)	56.38 (37.77-109.11)	0.177^b^
HOMA1-s (%)	23.08 (15.66-36.10)	23.84 (17.07-39.49)	0.437^b^	24.39 (10.56-43.68)	31.95 (13.67-40.03)	0.271^b^
HOMA2-IR	1.75 (0.99-2.47)	1.78 (1.14-2.61)	0.386^b^	1.73 (0.96-3.63)	1.39 (1.05-3.13)	0.961^b^
HOMA2-b (%)	46.55 (34.80-82.30)	56.6 (35.10-101.05)	0.206^b^	46.55 (30.65-87.10)	51.20 (36.78-94.25)	0.096^b^
HOMA2-s (%)	56.90 (40.35-100.90)	56.05 (38.35-87.80)	0.253^b^	57.80 (27.50-103.97)	71.90 (32.08-95.35)	0.612^b^
Hs-CRP (ng/mL)	2994.59 (1390.82-4117.30)	2576.82 (1115.72-5173.15)	0.912^b^	2213.11 (884.43-3984.21))	2050.28 (603.10-3431.99)	0.683^b^


**Table3:** Comparison of changes of measured markers between groups after melatonin intervention trial

	**Change from baseline**	***p *** **value**
Melatonin(ng/L)		0.192^a^
Melatonin	3.38(1.33)	
Placebo	0.94(1.28)	
HbA1c (%)		0.912^a^
Melatonin	-0.11(0.15)	
Placebo	-0.08(0.14)	
FBG (Mg/dL)		0.897^a^
Melatonin	-13.80(4.88)	
Placebo	-14.68(4.74)	
Insulin(μU/mL)		0.359^b^
Melatonin	1.24 (-1.92-4.16)	
Placebo	0.17 (-3.26-3.38)	
HOMA1-IR		0.200^b^
Melatonin	0.28 (-1.24-1.74)	
Placebo	-0.51 (-1.76-0.81)	
HOMA1-b		0.934^b^
Melatonin	3.58 (-15.83-46.77)	
Placebo	4.85 (-7.90-23.29)	
HOMA1-s (%)		0.148^b^
Melatonin	-2.33 (-10.61-9.16)	
Placebo	2.33 (-3.59-12.46)	
HOMA2-IR		0.414^b^
Melatonin	0.141 (-0.35-0.50)	
Placebo	0.050 (-0.48-0.34)	
HOMA2-b(%)		0.953^b^
Melatonin	2.90 (-16.20-44.62)	
Placebo	4.85 (-3.70-20.15)	
HOMA2-s(%)		0.164^b^
Melatonin	-5.35 (-18.82-9.75)	
Placebo	-0.15 (-8.85-14.72)	
Hs-CRP (ng/mL)		0.760^b^
Melatonin	17.50 (-1225.38-1171.82)	
Placebo	-38.63 (-526.02-626.53)	

